# Transstadial Effects of *Bti* on Traits of *Aedes aegypti* and Infection with Dengue Virus

**DOI:** 10.1371/journal.pntd.0004370

**Published:** 2016-02-12

**Authors:** Barry W. Alto, Cynthia C. Lord

**Affiliations:** University of Florida, IFAS, Department of Entomology and Nematology, Florida Medical Entomology Laboratory, Vero Beach, Florida, United States of America; North Carolina State University, UNITED STATES

## Abstract

Most mosquito control efforts are primarily focused on reducing the adult population size mediated by reductions in the larval population, which should lower risk of disease transmission. Although the aim of larviciding is to reduce larval abundance and thus recruitment of adults, nonlethal effects on adults are possible, including transstadial effects on phenotypes of adults such as survival and pathogen infection and transmission. In addition, the mortality induced by control efforts may act in conjunction with other sources of mosquito mortality in nature. The consequences of these effects and interactions may alter the potential of the population to transmit pathogens. We tested experimentally the combined effects of a larvicide (*Bacillus thuringiensis ssp*. *israelensis*, *Bti*) and competition during the larval stages on subsequent *Aedes aegypti* (Linnaeus) traits, population performance, and susceptibility to dengue-1 virus infection. *Ae*. *aegypti* that survived exposure to *Bti* experienced accelerated development, were larger, and produced more eggs with increasing amounts of *Bti*, consistent with competitive release among surviving mosquitoes. Changing larval density had no significant interactive effect with *Bti* treatment on development and growth to adulthood. Larval density, but not *Bti* or treatment interaction, had a strong effect on survival of adult *Ae*. *aegypti* females. There were sharper declines in cumulative daily survival of adults from crowded than uncrowded larval conditions, suggesting that high competition conditions of larvae may be an impediment to transmission of dengue viruses. Rates of infection and dengue-1 virus disseminated infections were found to be 87±13% and 88±12%, respectively. There were no significant treatment effects on infection measurements. Our findings suggest that larvicide campaigns using *Bti* may reduce the number of emerged adults, but survivors will have a fitness advantage (growth, development, enhanced production of eggs) relative to conspecifics that are not under larvicide pressure. However, under most circumstances, these transstadial effects are unlikely to outweigh reductions in the adult population by *Bti* and altered risk of disease transmission.

## Author Summary

Effective control of mosquito-borne diseases like dengue fever has historically been achieved by controlling vector populations. The use of larvicides that kill the larval stages of the yellow fever mosquito is a critical component of effective control. However, larvicides may act together with other components of the environment like larval crowding and alter the biology of adult mosquitoes. The authors found that mosquitoes that survived exposure to the larvicide *Bacillus thuringiensis spp*. *israelensis* (*Bti*) experienced faster development, were bigger, and produced more offspring. Larval crowding, but not larvicide *Bti*, had a strong effect on survival of adult *Aedes aegypti* (Linnaeus) females. Adult mosquitoes from crowded larval conditions had reduced survival compared to individuals from uncrowded larval conditions. Exposure to the larvicide *Bti* or larval crowding did not alter the ability of the surviving adult mosquitoes to become infected with dengue virus. Our findings suggest that larvicide campaigns may reduce the number of emerged adults, but survivors will have a fitness advantage (growth, development, enhanced production of offspring) relative to mosquitoes not exposed to the larvicide. However, under most circumstances, these effects are unlikely to outweigh reductions in the adult population by larvicide *Bti* and altered risk of disease transmission.

## Introduction

*Aedes aegypti* (Linnaeus) is regarded as one of the most important vectors of arboviruses that affect human health, including yellow fever, chikungunya, and dengue. Protecting humans from the diseases transmitted by this mosquito has historically been achieved by controlling mosquito populations. In conjunction with the use of pesticides, development and testing of non-pesticide control strategies and products is ongoing to determine their utility for the control of mosquito vectors of disease (e.g., genetically modified organisms, [1]; *Wolbachia* symbionts, [2]). Control approaches to reduce populations of *Ae*. *aegypti* have focused on the use of larvicides (e.g., temephos, *Bacillus thuringiensis spp*. *israelensis* (*Bti*), methoprene), space spraying (aircraft and vehicle-mounted ULV sprayers and portable thermal foggers of insecticides such as malathion and pyrethroids), source reduction, biological control (larvivorous fish and copepods), and education of the public [3, 4]. Despite the heavy reliance on larval control, there is little understanding of how larval control interventions (insecticide, biological) act in conjunction with other sources of larval mortality in nature to influence population size of adults, characteristics of surviving adult mosquitoes and subsequent consequences for risk of disease transmission.

Density-dependent processes which induce mortality of mosquito vectors during the immature stages also influence recruitment to the adult stage as well as growth and development [5]. Density-dependence attributable to larval competition has been demonstrated for mosquito vectors of pathogens (e.g. *Ae*. *aegypti*, [6]; *Culex quinquefasciatus* Say, [7]; *Anopheles gambiae* Giles, [8]) and extends to other mosquito species (e.g., [9]). Specifically, there are greater numbers of larvae that die when there are many larvae than when there are few conspecific larvae, attributable in most instances to competition for food resources. Additionally, density-dependent size at metamorphosis may be the basis for population regulation in some mosquito species (*Aedes sierrensis* Ludlow) [10].

Density-dependent competition may also have other consequences on risk of disease transmission, such as changes in characteristics of the surviving adult mosquitoes. In other words, density-dependence in the larval stages may have transstadial effects that are realized in the adult stage. Alterations in adult traits or population genetics associated with biting behavior, adult survival, and vector competence for pathogens among survivors after density-induced mortality events could influence vectorial capacity. For example, inter- and intraspecific competition among container mosquitoes alter adult survival [11–13] and vector competence for arboviruses [14–16].

It is often assumed that larval control practices which cause mortality among juvenile mosquitoes will act additively with other sources of mortality resulting in lower adult population size. Although this may be the case in most instances, alternative outcomes are possible [9, 17]. For example, larval competition can be severe in container inhabiting mosquitoes, such that many individuals die in the larval stage. If competition is reduced (a lower number of larvae in a container), more individuals may survive to adulthood [18]. Results observed in other systems have demonstrated that competitive stress may enhance [19, 20] or diminish [21] the lethal effects of pesticides [19, 20], hypothesized to be caused in part through changes in the food web. So, sources of mortality may have additive or non-additive effects. Additive refers to the arithmetic sum of individual effects, whereas non-additive refers to departure from additivity. If multiple causes of mortality are additive, total mortality is the sum of mortality from each source. Non-additive mortality may be observed in compensatory mortality, where the total number surviving is not affected by multiple mortality causes, or in overcompensation, where multiple causes of mortality result in lower total mortality than if acting alone [22]. Therefore, control measures aimed at reducing the larval population can have unanticipated outcomes on the adult numbers as well as characteristics of the adult survivors. Assessing the ultimate consequences of changes in larval density to the final population size or potential for pathogen transmission requires knowledge of the individual and combined effects.

Exposure to pesticides during the larval stages can alter immunity which may influence susceptibility to pathogens and infectivity [23–26]. Insecticides like *Bti* may influence mosquito life history traits and susceptibility to pathogens through changes in microbial communities and nutrient dynamics [27] and by changes in the energy budget and immune system. For example, exposure to an organophosphate during the larval stages altered the expression of immune-related genes in larvae and adult female *Ae*. *aegypti* [28]. Additionally, insecticide resistance is energetically costly, including resistance to *Bti* [29], which may compromise immune responses (e.g. production of detoxification enzymes that result in metabolic resistance [30, 31], and interfere with pathogen infection [32]. The effect of intraspecific competition among larvae and exposure to the organophosphate malathion on *Ae*. *aegypti* and *Aedes albopictus* (Skuse) survivorship to adulthood and life history traits among survivors have been examined using Sindbis virus as a model system [28, 33]. For both species, competition and the presence of malathion reduced survival to adulthood. The pesticide reduced larval density, eliminating the negative effects of competition that otherwise resulted in lengthened development time and small adults. Thus, malathion treatment led to short development time and large adults in high competition. For *Ae*. *aegypti*, but not *Ae*. *albopictus*, high competition conditions and the presence of malathion led to an increase in the number of mosquitoes with disseminated Sindbis virus infections [33]. These results suggest that competition and pesticides may influence disease transmission directly by altering recruitment to the population of adults and indirectly by altering phenotypes of adults (size, competence for arboviruses). Although these initial studies suggest an indirect role of malathion on mosquito competence using a model arbovirus system, there is a need to evaluate these effects for pathogens important to human health [34]. The current study investigates the relationship between density-dependence and *Bti*, one of the pesticides currently used to control dengue vectors [35, 36].

This paper reports on laboratory investigations to test *Bti* and density-dependent effects on *Ae*. *aegypti* traits, population performance, and infection with dengue-1 virus. Our first goal was to determine whether *Bti* affects adult survival and reproduction. Secondly, we investigated whether *Bti* interacts with intraspecific competition to alter adult survival, propensity to blood feed and barriers to infection and dissemination for dengue-1 virus. We explore possible effects using multiple levels within each *Bti* and density treatment. The estimates obtained from these experiments will facilitate parametrization of models aimed at investigating the interactive effects of larval crowding and *Bti* on risk of dengue transmission.

## Materials and Methods

### Mosquitoes

Mosquitoes used were F_3_ progeny of larvae collected from Key West, Florida. We used *Ae*. *aegypti* from Key West as this population vectored dengue virus in 2009 and 2010. Larvae were hatched from eggs submerged in containers with 1.0 L tap water and 0.15 g of larval food consisting of an equal mixture of brewer’s yeast and lactalbumin at 25±1°C and a 14:10 L:D photoregime. First instar larvae were rinsed free of larval food and added to experimental microcosms consisting of 2.5 L cylindrical plastic containers with lids, 2.0 L tap water and 0.2 g larval food. No additional food was added during the experiments. The experimental containers were maintained under the same temperature and photoregime used during larval hatching.

### Influence of *Bti* on immature development and adult survival and reproduction

Each experimental container was stocked with 300 newly hatched *Ae*. *aegypti* larvae. *Bti* was applied to each replicate container on the same day the larvae were added. The concentrations of *Bti* applied were 0 (control), 0.0009, 0.0025, 0.007, 0.0194, 0.054, 0.15, and 0.25 parts per million (ppm) using our own dilutions from a commercially available formulation with a potency of 3000 International toxic units (ITU) per mg. The levels of *Bti* used approximate the LD50 (LD50 for a similar *Bti* product, VectoBac WG: active ingredient: 3,000 *Bti* ITU per mg, 0.017–0.018 ppm for 3^rd^ instar *Ae*. *aegypti* [37], as well as concentrations below and above the LD50. No mosquitoes survived to adulthood in concentrations of 0.0194 ppm or higher. We replicated treatments with exposure to *Bti* three times, whereas treatments not exposed to *Bti* (0 ppm, control) were replicated four times. Experimental containers were maintained in a walk-in incubator. On a daily basis experimental containers were examined for pupae which were transferred to plastic vials plugged with cotton to capture emerged adults. The position of treatment containers within the incubator were changed daily. Newly eclosed adult *Ae*. *aegypti* were recorded by sex and date and then transferred to 16 ounce (by volume) cylindrical cages. Mosquitoes were provided with a 20% sucrose solution. Adult females aged 7–10 days were allowed to feed on defibrinated bovine blood using an artificial feeding system (Hemotek, Discovery workshops, Accrington, UK). Fully engorged females were separated from unfed and partially fed mosquitoes and held individually in cages. Mosquitoes that took partial blood meals were used in calculation of development and survivorship to adulthood. However, these mosquitoes were not used in calculation of adult survival, fecundity, and size. Each cage contained a plastic cup (30ml volume) filled with water and lined with seed germination paper as an oviposition substrate. Fully engorged females were maintained using the same environmental conditions and access to water and sucrose. Mosquitoes were monitored daily and the date of death was recorded by treatment and replicate. As an indicator of body size, the wings of mosquitoes were dissected and measured by length (axillary incision to wing tip) from a photo using a microscope and image analysis software (Media Cybernetics, Maryland, USA). The number of eggs oviposited by individual females during the first gonotrophic cycle was counted. For each container replicate we used all the mosquitoes available to measure survivorship to adulthood, development time, size, number of eggs oviposited, and adult survival.

### Effect of *Bti* and competition on adult survival and infection with dengue-1 virus

#### Density and *Bti* treatments

The experiment employed a factorial combination of five intraspecific larval densities and four *Bti* concentrations. Each experimental container was stocked with initial densities; 50, 100, 150, 250, and 300 (0.025–0.15 larvae/ml). Larval densities were within natural densities in Florida containers occupied by *Ae*. *aegypti* and competitor *Ae*. *albopictus* (N = 790, mean±SE, 0.17±0.02, range 0.00083–3.08 larvae/ml [14]. *Bti* was applied to each replicate container on the same day the larvae were added. The concentrations of *Bti* applied were 0 (control), 0.0042, 0.0056, and 0.007 ppm. We replicated treatments with exposure to *Bti* three times and treatments not exposed to *Bti* (0 ppm, control) were replicated four times. Experimental containers were maintained similarly as in the first experiment. Adult females from this experiment were then used in an infection experiment with dengue-1 virus (DENV-1).

#### Estimated finite rate of increase (λ')

An estimated finite rate of increase (λ') was calculated for each experimental container;

$$\lambda^{\prime }=\text{exp}\left(r^{\prime }\right)=\text{exp}\left[\frac{\text{ln}\left[\left(\frac{1}{N_{0}}\right)\sum_{x}A_{x}f(w_{x})\right]}{D+\left[\frac{\sum_{x}xA_{x}f(w_{x})}{\sum_{x}A_{x}f(w_{x})}\right]}\right]$$

where *N*_*0*_ is the initial number of females in the cohort (assumed to be 50%); *A*_*x*_ is the number of females eclosing on day x; *w*_*x*_ is the mean adult female size on day *x*, and D is the time from eclosion to reproduction taken as 12 d [38]. The relationship between size and fecundity was *f* (*w*_*x*_) = 60.730(*w*_*x*_*)*– 111.76 based on mosquitoes from our first experiment.

#### Virus

Dengue-1 virus (GenBank accession number JQ675358) was obtained from a human in Key West, FL during an outbreak of dengue and subsequently passaged twice in Vero cells prior to use in the infection study. Viruses and host cells were cultured in media containing medium 199, 10% fetal bovine serum, 100 units/mL of penicillin, and 100 μg/mL of streptomycin.

#### Oral infection of mosquitoes

Infected blood meals were prepared by harvesting media from monolayers of Vero cells previously inoculated with a multiplicity of infection of 0.01 (viruses/cell) seven days prior. Virus infected media were combined with an equal volume of defibrinated bovine blood. Adult females aged 7–10 days were allowed to feed on DENV-1 infected blood using an artificial feeding system. Fully engorged females were separated from unfed and partially fed mosquitoes and held at 28°C with access to 20% sucrose solution. After 14 days of incubation, mosquitoes were stored at -80°C. Blood feeding rates were calculated as the number of fully engorged mosquitoes from the total of mosquitoes offered blood. Mosquitoes that did not blood feed were maintained under similar conditions and monitored daily until death.

#### Determination of susceptibility to virus infection and dissemination

Mosquitoes were dissected to remove legs and a single wing from the remainder of the body. Bodies and legs were triturated separately in centrifuge tubes with 0.9 mL BA-1 (10x medium 199, 1% bovine serum albumin, 0.05 M TRIS, 100 units/mL of penicillin, 100 μg/mL of streptomycin, 1 μg/mL of mycostatin). Nucleic acid was extracted from 250-μL samples and eluted in 50 μL buffer using the MagNA Pure LC Total Nucleic Acid Isolation Kit (Roche Diagnostics, Indianapolis, IN). Viral RNA in samples was determined using the Superscript III One-Step Quantitative RT-PCR System (Invitrogen, Carlsbad, CA) with a Light Cycler 480 system (Roche, Mannheim, Germany) [39]. A standard curve method was used to relate the amount of DENV RNA present in mosquito samples to serial dilutions of virus stock with known concentrations expressed in plaque forming unit equivalents (pfue)/ml [40, 15]. Infection rate was calculated as the percent of mosquitoes with DENV-1 RNA present in their bodies from the total number that fed. Dissemination rate was calculated as the percent of mosquitoes with infected bodies that have DENV-1 RNA present in their legs. A total of 755 adult females were used in testing for DENV-1.

#### Statistical analysis

Treatment effects on survivorship to adulthood, female size, and female development were analyzed using ANOVA and multivariate analysis of variance (MANOVA). Standardized canonical coefficients (SCC) were used to determine the relative contribution of survivorship, size, and development when significant treatment effects were detected (PROC GLM, SAS 9.22). Blood feeding rates, number of eggs oviposited, and λ' were analyzed by ANOVA. When significant treatment effects were detected, pair-wise contrasts of means were performed (PROC GLM, SAS 9.22). Regression analysis was used to relate mosquito wing length to number of eggs oviposited (PROC REG, SAS 9.22).

Treatment effects on survival of adults were compared using a regression analysis of survival data based on the Cox proportional hazards model (PROC PHREG, SAS 9.22). Treatment effects on susceptibility to DENV-1 infection and dissemination were analyzed using MANOVA and SCC. Treatment effects on DENV-1 titer in the bodies and legs of mosquitoes were analyzed using ANOVA.

## Results

### Influence of *Bti* on immature development and adult survival and reproduction

**Fig 1:**
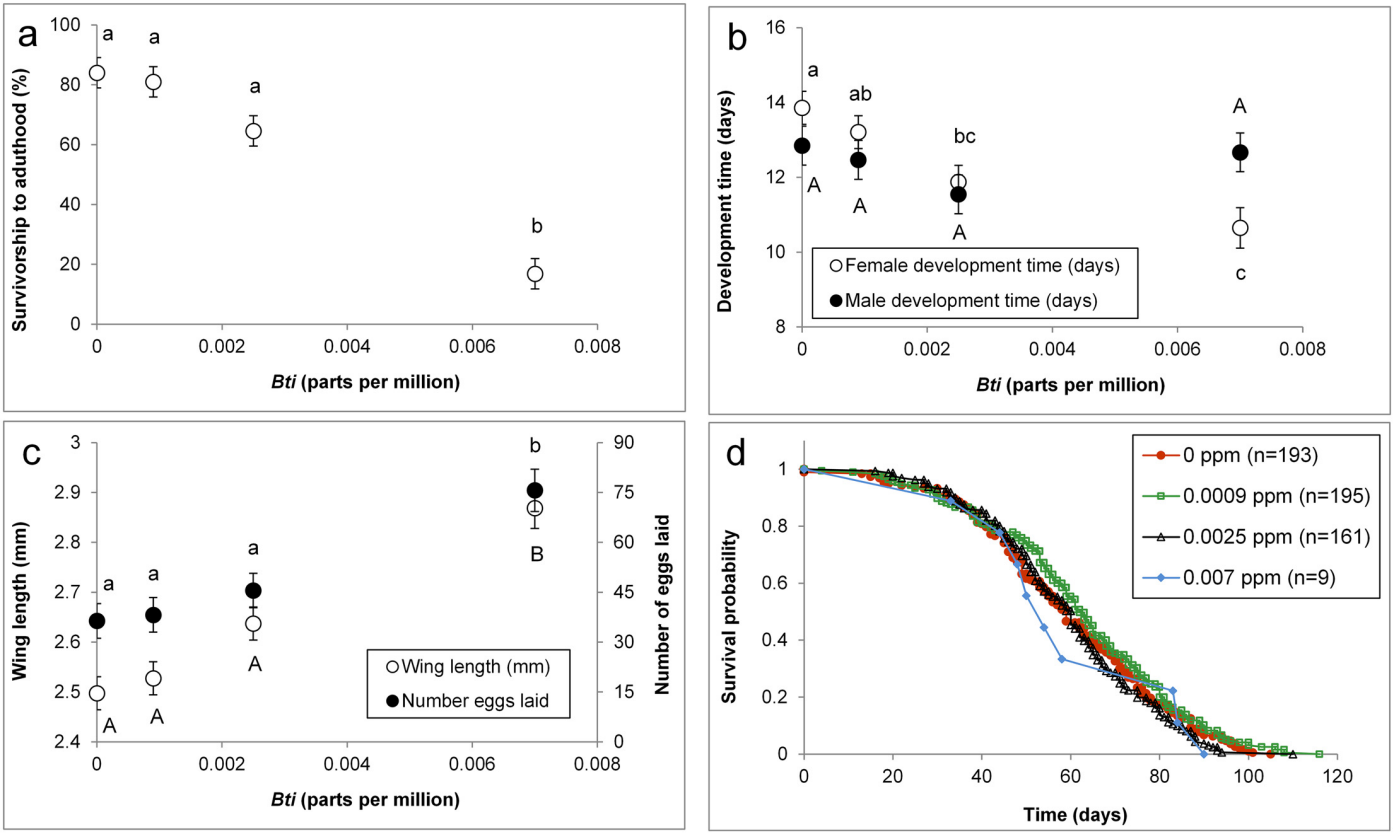
*Bacillus thuringiensis israelensis* treatment effects on *Aedes aegypti* a) survivorship to adulthood, b) female and male development time, c) female wing length and number of eggs oviposited, and d) adult female survival. Symbols associated with different letters show significant differences. The number of mosquitoes tested is denoted in parentheses. There were no survivors at *Bti* concentrations ≥0.0194.

Mean survivorship to adulthood was significantly affected by the concentration of *Bti* (*F*_7,16_ = 58.01, *p*<0.0001), with no survivors at concentrations ≥ 0.0194. Increasing concentrations of *Bti* were associated with significantly lower survivorship to adulthood (Fig 1a). Development time of females, from treatments with survivors, was significantly affected by the presence of *Bti* (*F*_3,7_ = 8.48, *p* = 0.009). In the absence of *Bti*, development times were significantly longer than treatments with *Bti*, except at the lowest level of *Bti*. In the presence of *Bti*, there was a consistent decrease in development time of females with increases in the concentration of *Bti* (Fig 1b). Development time of males were unaffected by the presence of *Bti* (*F*_3,8_ = 1.20, *p* = 0.36; Fig 1b). Regression analysis showed a significant positive relationship between *Ae*. *aegypti* wing length (mm) and number of eggs oviposited (y = 60.737x-111.76, r^2^ = 0.35, n = 212, p<0.0001; where y = number of eggs and x = wing length in mm). Significantly longer wing lengths and more eggs were oviposited by *Ae*. *aegypti* from the highest *Bti* treatment than all other treatments (*F*_3,7_ = 9.11, *p* = 0.008; Fig 1c). The relationship between wing length and number of eggs oviposited did not differ between *Bti* treatments (test for equal slopes, F_3_ = 1.17, *p* = 0.32). Thus, the slopes were not different between *Bti* treatments. There was an approximate 46% increase in mean number of eggs oviposited by females exposed to the highest amount of *Bti* relative to the control. A caveat to this interpretation is that females were not dissected and examined for retained eggs, thus potentially underestimating fecundity. A total of 558 adult females were monitored daily to record date of death, which was used to establish treatment dependent survival distributions. Survival analyses showed no significant differences in adult survival among *Bti* treatments (χ^2^ = 5.13, df = 3, *p* = 0.16; Fig 1d).

### Effect of *Bti* and competition on adult survival and infection with dengue-1 virus

#### Size, development, survivorship and λ'

**Fig 2:**
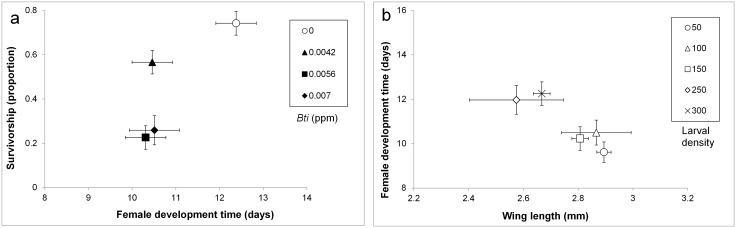
Bivariate plots of least-squares means (±SE) for three dependent variables for *Aedes aegypti* females. a) The effects of pesticide treatment on survivorship to adulthood and female development time. b) The effects of density treatment on wing length and female development time.

MANOVA showed significant effects of pesticide and density treatments on survivorship to adulthood, development, and size. The interaction between these two factors was not significant (Table 1). SCCs showed that differences in survivorship to adulthood and female development time contributed the most to the significant effect of pesticide. Increases in pesticide were associated with decreased survivorship and shorter development times (Fig 2a). SCCs showed that differences in wing length and female development time contributed the most to the significant effect of density (Table 1). Increases in density were associated with shorter wing lengths and lengthened development times (Fig 2b).

**Table 1:** Multivariate analysis of variance and standardized canonical coefficients (SCC) for treatment effects of density, presence of pesticide (*Bti*) and interaction on survivorship to adulthood, female development time and female wing length.

Source	df (num., denom.)	Pillai’s trace	*p*^1^		SCC	
				survivorship	development	wing length
**Pesticide (P)**	9, 108	0.68	**0.0007**	1.19	0.52	-0.10
**Density (D)**	12, 108	0.94	**<0.0001**	0.27	-0.47	1.48
**P x D**	30, 108	0.53	0.77	0.13	-0.25	1.57

^1^Significant *p*-values are in bold.

**Fig 3:**
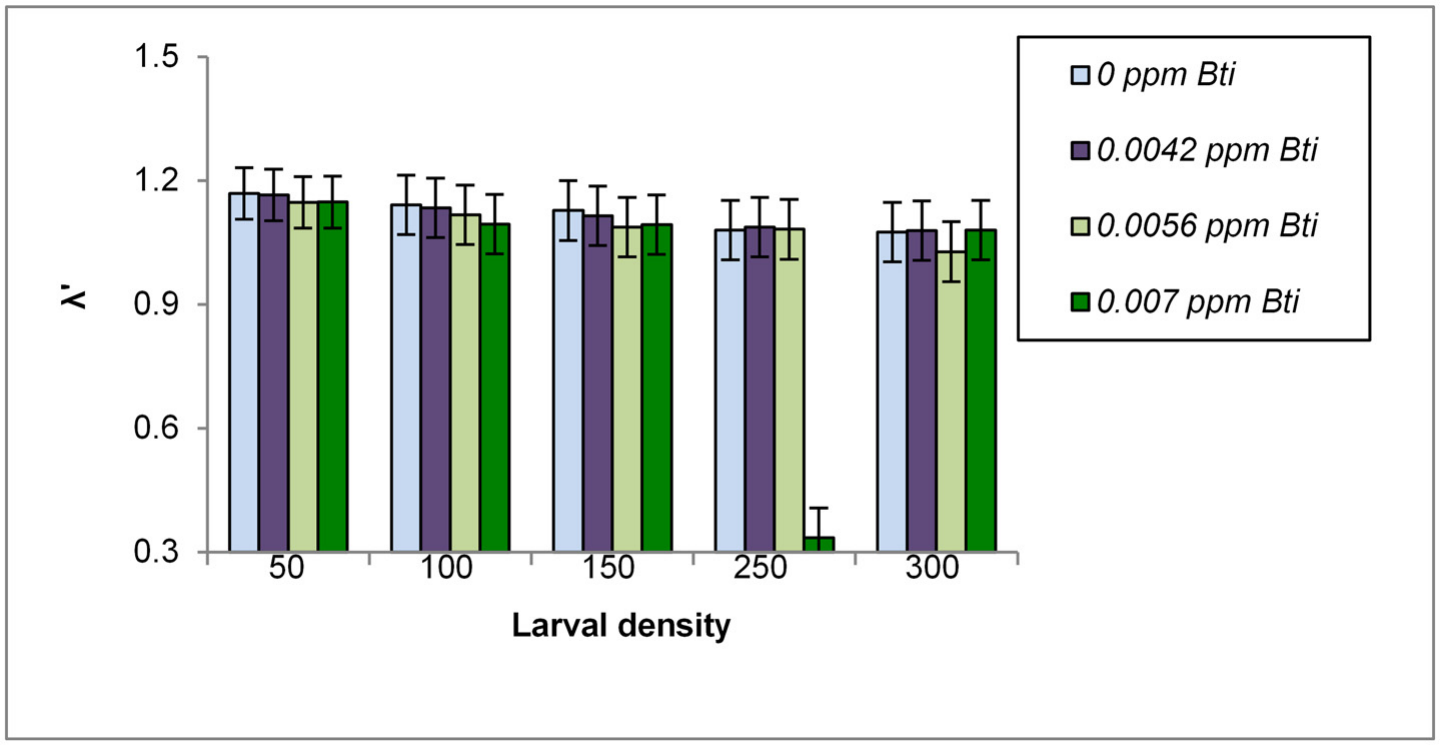
Least-squares means (±SE) for λ' for *Aedes aegypti* from *Bacillus thuringiensis israelensis* and density treatments.

ANOVA showed significant effects of pesticide, density, and interactions on λ' (all F≥6.50, p≤0.001). The interaction was attributable to significantly lower λ'-values from the highest *Bti* treatment and a density of 250 initial larvae (mean±SE, 0.33±0.07) than all other treatments. All remaining pairwise comparisons of treatments did not significantly differ from one another. λ' in the highest *Bti* treatment was significantly lower than in all other treatments (Fig 3). λ' values were not significantly different between the other *Bti* treatments. λ' in the 250 initial larvae treatment was significantly lower than in all other density treatments (Fig 3). The reason for the relatively low values of λ' associated with the highest *Bti* treatment and a density of 250 initial larvae is because two of the three replicates failed to produce adult females, thus yielding zero values for λ' for these replicates.

#### Adult blood feeding and survival

Treatment effects of density, pesticide, and their interaction on the immature stages did not significantly affect blood feeding rates of adult females (all F<1.51, p>0.18; Table 2, mean±SE, 59.8±17.5%).

**Table 2:** Analysis of variance for larval density and pesticide treatment effects on rate of blood feeding. Regression analysis of survival of adult *Aedes aegypti* females in response to treatment effects of density, pesticide (*Bti*) and their interaction.

		Blood feeding			Survival of adults	
Source	df	F	*p*^1^	df	*χ*^*2*^	*p*^1^
**Density (D)**	3	1.37	0.27	4	37.20	**<0.0001**
**Pesticide (P)**	4	0.70	0.60	3	5.77	0.12
**D x P**	10	1.51	0.18	11	15.71	0.15

^1^Significant *p*-values are in bold.

**Fig 4:**
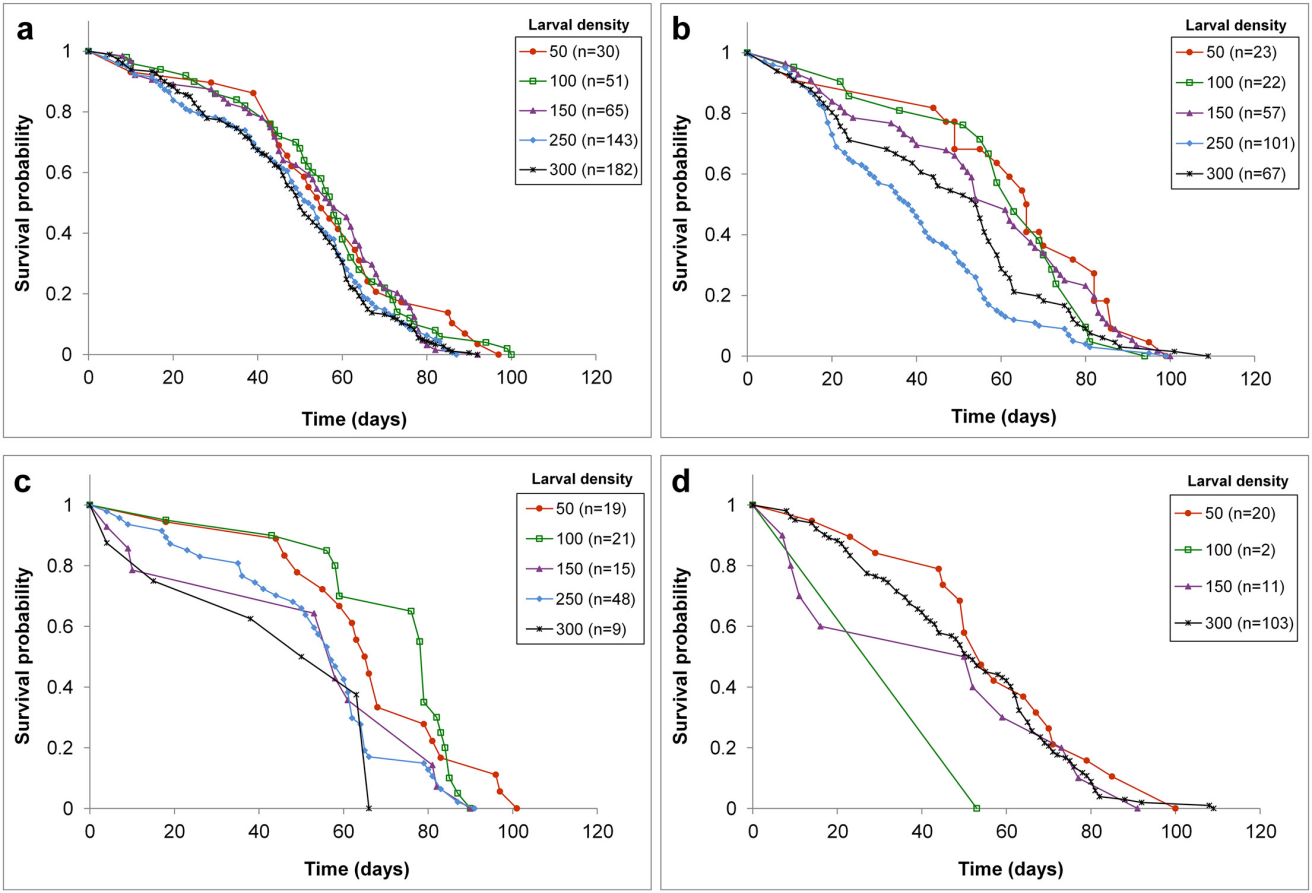
Density and treatment effects on *Aedes aegypti* adult female survival (969 mosquitoes tested) derived from immature environments with *Bacillus thuringiensis israelensis* concentrations of a) 0 parts per million (ppm), b) 0.0042 ppm, c) 0.0056 ppm, and d) 0.007 ppm. Larval densities range from 50–300 and the number of mosquitoes tested is denoted in parentheses. The survival of females is not shown for 0.007 ppm *Bti* and 250 density of *Ae*. *aegypti* because there were too few survivors.

A total of 969 adult females were used to establish treatment dependent survival distributions. Survival of adult females depended on density but not pesticide or the treatment interaction (Table 2). The treatment 250 larvae and 0.007 ppm *Bti* yielded very few survivors, thus limiting our ability to make pairwise comparisons with the analysis that included both *Bti* and density treatments. Because the effects of *Bti* were not significant, we reduced the model to test only for density, enabling us to perform all possible pair-wise comparisons for the density treatment. Comparisons of survival over time showed significantly steeper declines at initial densities of 300, 250, and 150 compared to 50 and 100 *Ae*. *aegypti* larvae (Table 3 and Fig 4). Survival did not significantly differ among adult females produced from the three highest density larval environments at various *Bti* concentrations.

**Table 3:** Pairwise comparisons for significant density treatment effect on adult *Aedes aegypti* female survival. P-values were corrected for multiple comparisons using the Tukey-Kramer method.

Comparison	Estimate	Std. Error	z value	Adjusted *p*^1^
**100 vs 150**	-0.14	0.13	-1.08	0.816
**100 vs 250**	-0.53	0.12	-4.45	**<0.0001**
**100 vs 300**	-0.41	0.11	-3.53	**0.0038**
**100 vs 50**	0.07	0.14	0.52	0.985
**150 vs 250**	-0.39	0.10	-3.81	**0.0013**
**150 vs 300**	-0.27	0.09	-2.72	**0.050**
**150 vs 50**	0.22	0.13	1.63	0.477
**250 vs 300**	0.12	0.07	1.51	0.554
**250 vs 50**	0.61	0.12	4.97	**<0.0001**
**300 vs 50**	0.49	0.12	4.09	**0.0004**

^1^Significant *p*-values are in bold.

#### Susceptibility of *Ae*. *aegypti* for dengue-1 virus

**Fig 5:**
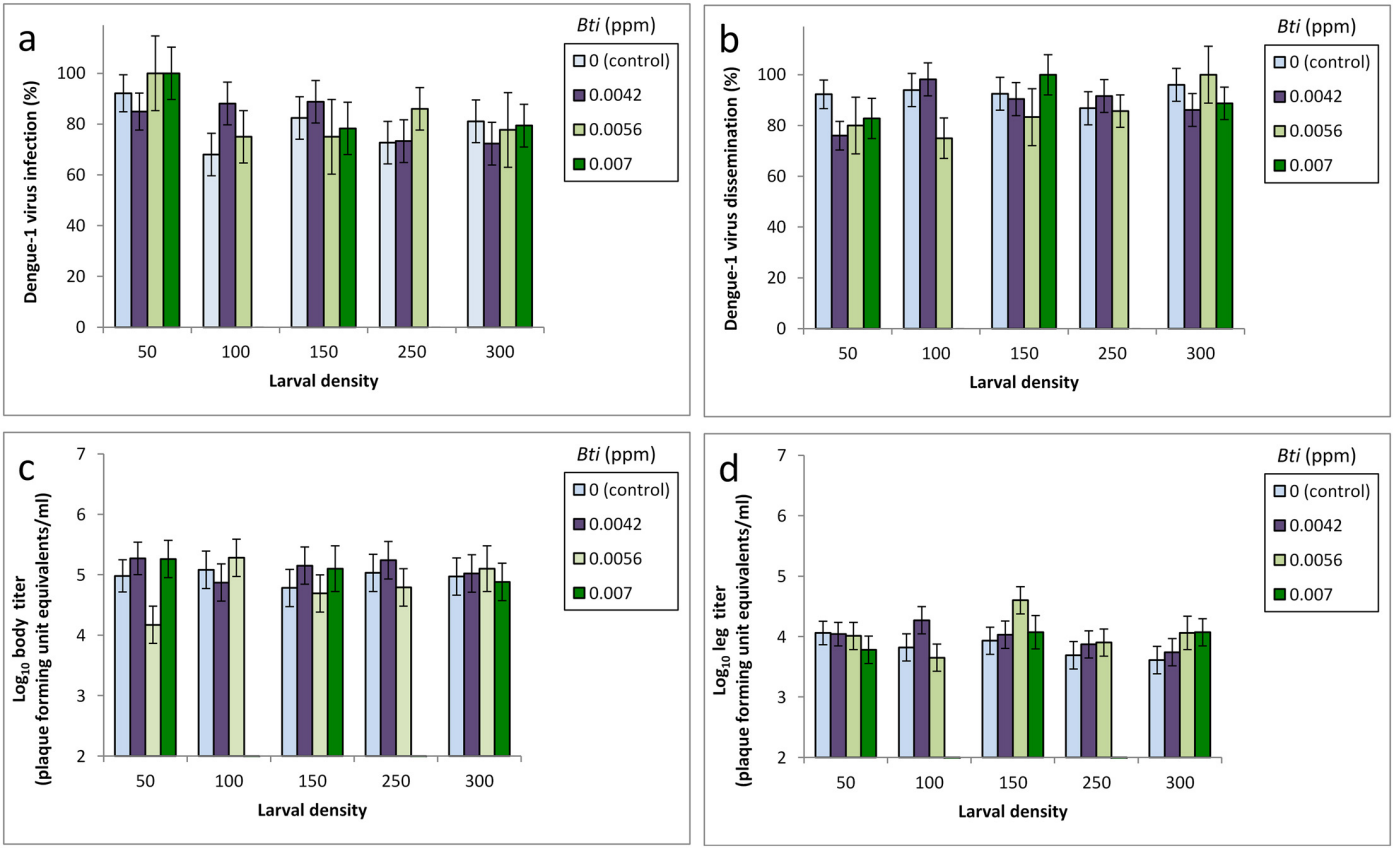
Least-squares means (±SE) for treatment effects of Bacillus thuringiensis israelensis and initial larval density on Aedes aegypti a) body infection, b) leg infection (viral dissemination), c) body viral titer (plaque forming unit equivalents/ml), and d) leg viral titer (plaque forming unit equivalents/ml) following exposure to dengue-1 virus.

The dengue viral titers in blood meals were within the range of viremia in humans (mean±SD, 7.2±0.8 PFU/ml [41, 42]. A total of 755 adult females were tested for susceptibility to infection and viral dissemination for dengue-1 virus. Rates of infection and viral dissemination were 87±13% and 88±12%, respectively (Fig 5a and 5b). Dengue-1 virus body and leg titers were 4.9±0.5 and 3.9±0.2 log10 plaque forming unit equivalents/ml (Fig 5c and 5d). Treatment effects of density, pesticide, and their interaction experienced by the immature stages did not significantly affect infection and viral dissemination rates as well as viral titer of adult females (Table 4).

**Table 4:** Multivariate analysis of variance and standardized canonical coefficients (SCC) for treatment effects of density, presence of pesticide (*Bti*) and interaction on infection and viral dissemination rates, and viral titer in mosquito bodies and legs. Mean±SD provided for infection (%), dissemination (%), body titer (B), leg titer (L). Titers are expressed as log10 plaque forming unit equivalents/ml. A total of 755 adult females were used in testing for dengue-1 virus.

Source	df (num., denom.)	Pillai’s trace	*p*		SCC		
				Infection	Dissemination	Titer (B)	Titer (L)
**Pesticide (P)**	12, 90	0.31	0.58	0.38	-0.43	-0.06	1.02
**Density (D)**	16, 124	0.51	0.37	0.96	-0.51	-0.36	0.16
**P x D**	40, 124	0.97	0.48	0.25	0.46	-0.08	0.89
**Mean±SD**				87±13%	88±12%	4.9±0.5	3.9±0.2

## Discussion

Our results demonstrate that the outcome of mortality and phenotypic traits induced by *Bti* control efforts would not be expected to change at different amounts of larval crowding of *Ae*. *aegypti*. Female mosquitoes that survived exposure to *Bti* experienced accelerated development, were larger, and produced more eggs during the first gonotrophic cycle after being exposed to the highest concentration of *Bti*. Superior reproductive potential among survivors may facilitate recovery of local populations of *Ae*. *aegypti* following larviciding [18, 43]. Mosquito larvae in nature may be exposed to sublethal concentrations of larvicide attributable to recolonization of container habitats with sublethal amounts of larvicide or exposure to less than the “full” lethal dose at time of widespread application of larvicides (low-volume area-wide strategy) [44]. For example, changes in life history traits in our experiments occurred at high exposure to *Bti* that resulted in ~80% mortality (inhibition of adult emergence) which approximates observations of rates of mortality (87%) of area-wide ground applications of *Bti* to control dengue vector *Ae*. *albopictus* in residential neighborhoods [44]. Larvicide resistance in many populations of *Ae*. *aegypti* is further evidence for the occurrence of exposure to sublethal concentrations of larvicides in nature [45–48].

Our observations suggest that *Ae*. *aegypti* larvae may respond to reductions in the number of conspecifics attributable to *Bti* killing a fraction of the larvae. It is possible that selective mortality due to *Bti* allowed for survival of some individuals with rapid growth and development (e.g., lethal concentration increases with larval development) [49]. However, it seems more likely that the mechanism relates to changes in density and availability of nutrition (microbial biomass) because consumption by mosquito larvae reduces digestible microorganisms [50]. Although we did not directly measure microbial populations, reductions in the number of larvae with associated increases in per capita nutrient resources are consistent with observed changes in growth and development. Other studies have shown strong and complex interactive effects of *Bti* on microbial communities and nutrient dynamics in microcosms [27]. Our observations support previous studies showing similar effects of pesticides on growth and developmental responses in *Ae*. *aegypti*, including fungal larvicide *Metarhizium anisopliae* (Metschn.) Sorokīn [43], neurotoxin spinosad [51], organophosphate malathion [28, 33, 52, 53], and botanical insecticides [54].

Most mosquitoes are sexually dimorphic and protandrous, where the latter here refers to the eclosion of males before females into a seasonal breeding population. Sex-specific development and size may explain why the developmental response of males (demonstrating canalization) differed from females along a gradient of *Bti*. Males are often smaller than females and develop more quickly. Competitive release among surviving larvae accelerated metamorphosis among females (21% reduction in development time in the highest amount of *Bti* than controls), whereas the same benefit was negligible among males with inherently shorter developmental time. Sex-specific reaction norms (development, size, survival, nutrient reserves) have been observed among several container dwelling mosquito species exposed to predators [55, 56] competitors [57, 58], and varying nutrition [59] during the immature stages.

Mosquitoes experienced a 46% increase in the number of eggs produced at the highest amount of *Bti* than controls, which covaried with size. This means that mosquitoes exposed to sublethal amounts of *Bti* did not incur a physiological cost of reproduction. Alternatively, any physiological cost of reproduction was outweighed by enhanced size (13% increase) following exposure to *Bti*, presumably associated with reductions in the number of larvae. A caveat to this interpretation is that we did not measure cumulative lifetime fecundity (net reproductive rate) and so we cannot assess whether there are costs of reproduction later in life (e.g., daily fecundity through lifespan) [60]. However, substantially fewer individuals would survive the second gonotrophic cycle assuming a mean probability of daily survival of 0.6–0.89 [61–63] and approximate duration of the gonotrophic cycle of three days in nature [64]. Size of *Ae*. *aegypti* females in our study were within the range and approximated the mean size observed in the field [65–67]. Fecundity increases with size of adult *Ae*. *aegypti* females [68–70] as in most insects [71]. However, the current study is one of a few demonstrating an indirect role of a pesticide enhancing the number of eggs produced by mosquitoes (e.g.,[51]).

The presence of *Bti* did not alter survival of adults and so *Bti* appears to selectively influence traits of adults. Lifespan and fecundity determine fitness and so the lasting effects of sublethal exposure to *Bti* are likely to minimally influence (perhaps increase) net reproductive rate. The influence of *Bti* on mosquito life history traits acted independently of those effects attributable to larval crowding, suggesting that competitively stressed mosquitoes respond similarly to this pesticide as do mosquitoes from less stressful conditions. This suggests that the efficacy of *Bti* in nature should be robust to spatial and temporal heterogeneity in the number of larvae in containers. Responses in population performance to treatments were less distinct than those observed for life history traits. λ' is heavily influenced by changes in survivorship to adulthood. Despite using a 6-fold difference in the initial density of larvae, survivorship to adulthood contributed less than that of development and growth effects, which in part, explains the lack of many treatment differences in λ'. Curiously, two of the three treatments at the highest *Bti* concentration and a density of 250 initial larvae failed to produce adult females which largely contributed to observed treatment differences. Although it is unclear what accounts for the observed effect, non-linearities in the relationship between density and mosquito responses have been observed in other container-dwelling *Aedes* species [11, 72]. The highest concentration of *Bti* significantly reduced λ', but lower concentrations did not. This result suggests that larvicidal controls that achieve less than about 75% mortality may not be effective at achieving substantial declines in *Ae*. *aegypti* populations. This interpretation agrees in general with transmission models of dengue that have found that substantial levels of larval control such as source reduction would be needed to reduce *Ae*. *aegypti* pupae per person in an environment [73].

Lengthened development and reduced growth were largely responsible for the density effect, suggesting that larval crowding and nutrition were not limiting factors that substantially influenced survivorship to adulthood. We observed strong transstadial effects of density with steeper declines in survival of adult *Ae*. *aegypti* females from crowded larval conditions, perhaps attributable to changes in larval biosynthesis of nutritive reserves [69, 74–76] or an additional stressor on the mosquito’s physiology [28, 77, 78]. Our observations support findings from laboratory studies that used manipulations of larval nutrition and crowding and measured survival of adult *Ae*. *aegypti* females [11, 13, 76]. Measurements of daily survival rates of *Ae*. *aegypti* in nature and the laboratory have used size as an indicator of relative nutrition and crowding experienced during the immature stages. [66] analyzed laboratory results in which adult survival of *Ae*. *aegypti* increases with size, but decreases at the largest sizes (i.e., non-linear relationship between size and longevity). Similarly, the relationship between adult parous rate, used to measure mosquito survival, and size among wild-caught *Ae*. *sierrensis* females was curvilinear [79]. Using the same approach, field studies in Thailand and Puerto Rico found no relationship between parity status and size of *Ae*. *aegypti* [67]. A mark-release-recapture field study in Brazil showed that laboratory reared large adult *Ae*. *aegypti* males, but not females, had a survival advantage relative to smaller individuals from a low food diet [62]. Field studies often assume that heterogeneity in size of mosquitoes is largely determined by nutrition or larval crowding, however other factors may be responsible as well (environmental temperature, genetics). Collectively, these studies suggest that ecological conditions that larvae experience may have a strong transstadial effect on daily survival rates of adult *Ae*. *aegypti* females.

In the current study, the heterogeneity in sizes of adult females was comparable to those observed in *Ae*. *aegypti* in the laboratory [80, 81], but narrower than the range in the field [66], where differences in dengue virus infection were observed. The reason for lack of heterogeneity in dengue virus infection in the current study is unclear. However, infection and viral dissemination rates in *Ae*. *aegypti* females were much higher than those observed in other studies [15, 80, 81]. Thus, dose-dependent effects of virus exposure to *Ae*. *aegypti* females may obscure more subtle effects attributable to ecological conditions experienced by larvae. For example, our ability to detect a treatment-induced increase, but not reduction, in infection and viral dissemination is limited due to the relatively high rates observed. Also, survivorship to adulthood was higher in the current study (41–57%) compared to other studies (31–36%) [15] that have observed density-dependent heterogeneity in dengue virus infection, suggesting less competitive stress. Previous studies have demonstrated that intra and interspecific larval competition and availability of nutrients enhanced susceptibility to dengue virus infection [15, 81, 82]. However these effects appear to be stronger for *Ae*. *albopictus* than *Ae*. *aegypti* [15]. Similarly, larval competition enhanced infection and viral dissemination of Sindbis virus in *Ae*. *albopictus*, but not *Ae*. *aegypti* [14], suggesting species-specific differences in barrier(s) to infection and tolerance for stress.

Given the prominent role that larvicides serve in *Ae*. *aegypti* control programs, there is a compelling need to understand how larvicides act in concert with other sources of mortality in larval habitats in nature. Here we show that low concentrations of a common larvicide had direct lethal effects on *Ae*. *aegypti* immatures and indirect effects on select life history traits. We would expect that larvicide campaigns may reduce the number of emerged adults, but survivors will have a lifetime fitness advantage (growth, development, number of eggs oviposited) relative to conspecifics. However, we did not find any evidence to suggest that exposure to *Bti* changes survival of adult females or their rates of infection with dengue virus, both components of vectorial capacity. Under most circumstances, these transstadial effects are unlikely to outweigh reductions in the adult population by *Bti* and altered risk of disease transmission.
